# Joining Forces against Antibiotic Resistance: The One Health Solution

**DOI:** 10.3390/pathogens12091074

**Published:** 2023-08-23

**Authors:** Eleonora Cella, Marta Giovanetti, Francesca Benedetti, Fabio Scarpa, Catherine Johnston, Alessandra Borsetti, Giancarlo Ceccarelli, Taj Azarian, Davide Zella, Massimo Ciccozzi

**Affiliations:** 1 Burnett School of Biomedical Sciences, University of Central Florida, Orlando, FL 32816, USA; catherine.johnston@ucf.edu (C.J.); taj.azarian@ucf.edu (T.A.); 2 Sciences and Technologies for Sustainable Development and One Health, University Campus Bio-Medico of Roma, 00128 Roma, Italy; giovanettimarta@gmail.com; 3 Instituto Rene Rachou Fundação Oswaldo Cruz, Belo Horizonte 31310-260, Minas Gerais, Brazil; 4 Department of Biochemistry and Molecular Biology, Institute of Human Virology and Global Virus Network Center, University of Maryland School of Medicine, Baltimore, MD 21201, USA; fbenedetti@ihv.umaryland.edu (F.B.); dzella@ihv.umaryland.edu (D.Z.); 5 Department of Biomedical Sciences, University of Sassari, 07100 Sassari, Italy; fscarpa@uniss.it; 6 National HIV/AIDS Research Center (CNAIDS), National Institute of Health, 00161 Rome, Italy; alessandra.borsetti@iss.it; 7 Department of Public Health and Infectious Diseases, Sapienza University of Rome, 00161 Rome, Italy; giancarlo.ceccarelli@uniroma1.it; 8 Unit of Medical Statistics and Molecular Epidemiology, Università Campus Bio-Medico di Roma, 00128 Roma, Italy

**Keywords:** antimicrobial resistance, antibiotic resistance, One Health

## Abstract

Antibiotic resistance is a significant global health concern that affects both human and animal populations. The *One Health* approach acknowledges the interconnectedness of human health, animal health, and the environment. It emphasizes the importance of collaboration and coordination across these sectors to tackle complex health challenges such as antibiotic resistance. In the context of One Health, antibiotic resistance refers to the ability of bacteria to withstand the efficacy of antibiotics, rendering them less effective or completely ineffective in treating infections. The emergence and spread of antibiotic-resistant bacteria pose a threat to human and animal health, as well as to the effectiveness of medical treatments and veterinary interventions. In particular, One Health recognizes that antibiotic use in human medicine, animal agriculture, and the environment are interconnected factors contributing to the development and spread of antibiotic resistance. For example, the misuse and overuse of antibiotics in human healthcare, including inappropriate prescribing and patient non-compliance, can contribute to the selection and spread of resistant bacteria. Similarly, the use of antibiotics in livestock production for growth promotion and disease prevention can contribute to the development of antibiotic resistance in animals and subsequent transmission to humans through the food chain. Addressing antibiotic resistance requires a collaborative One Health approach that involves multiple participants, including healthcare professionals, veterinarians, researchers, and policymakers.

## 1. Examining the Global Impact of Antibiotic Resistance

Antimicrobial resistance (AMR) in pathogenic bacteria can be defined in two ways: microbiologically and clinically. Microbiological resistance is determined by the presence of a genetically acquired or mutated resistance mechanism in the pathogen, which is classified as resistant, intermediate, or susceptible based on specific cut-off values in laboratory tests [1]. Clinical resistance is defined by the level of antimicrobial activity that is associated with a high likelihood of treatment failure. Specifically, it means that using a drug against which the pathogen was tested and proved susceptible is more effective than using a drug against which the pathogen was tested and proved resistant. The cut-off values for clinical resistance may vary depending on factors such as the site of infection or the dosage of the drug [2].

AMR in bacterial pathogens is a significant global concern associated with high rates of illness and mortality [3,4]. Several factors increase the demands for antibiotic prescriptions, as well as situations that create financial incentives from medication distribution, such as unregulated access and usage of over-the-counter drugs, can lead to inappropriate prescribing of antimicrobials. Both Gram-positive and Gram-negative bacteria have developed multidrug resistant patterns, leading to challenging and sometimes infections that are untreatable with conventional antimicrobials. In many healthcare settings, there is a lack of early identification of causative microorganisms and their susceptibility to antimicrobials in patients with serious infections, leading to the excessive and unnecessary use of broad-spectrum antibiotics [3,4]. Consequently, there has been a notable increase in emerging resistance. Coupled with poor infection control practices, resistant bacteria can easily be disseminated to other patients and the environment [3,4]. 

**Figure 1:**
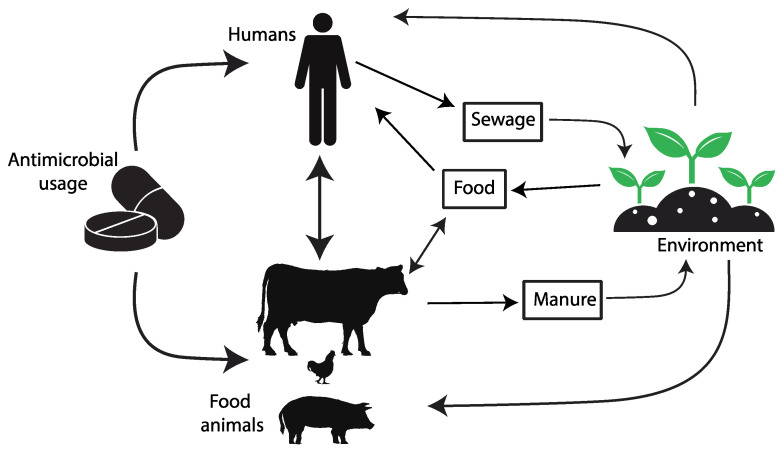
The diagram depicts the pathways of transmission for antimicrobial resistance between farm animals, the surrounding environment, and human populations. Image by https://www.rawpixel.com/ and https://www.vecteezy.com/ accessed on 26 July 2023).

The introduction of antibiotics as clinical agents radically changed the evolution and spread of resistance by providing considerable selective pressures, especially on members of the microbiota of humans and domestic animals but also in environments heavily polluted with antibiotics. This selective pressure has promoted the mobilization and horizontal transfer of a large range of antibiotic resistance genes (ARGs) among many bacterial species, particularly to those causing disease. The downstream consequences of such accumulating evolutionary events are gradually increasing the difficulty in the prevention and treatment of bacterial infections. As bacteria and mobile genetic elements often cross environments and species boundaries, it is critical to understand and acknowledge the connections between the human, animal, and environmental microbiota (the One Health Concept [5,6]) (https://www.cdc.gov/onehealth/ accessed on 1 July 2023) to manage this global health challenge [7,8,9,10,11,12,13,14,15,16,17] (Figure 1).

**Figure 2:**
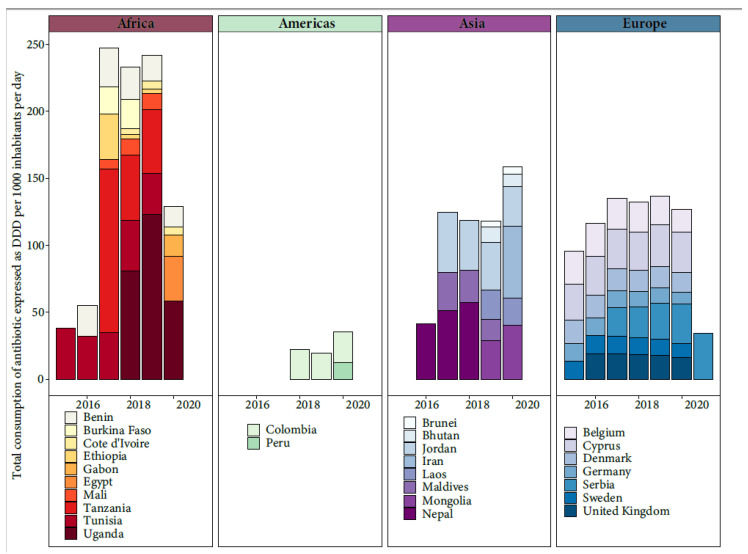
Total consumption of antibiotic expressed as Defined Daily Doses (DDD) per 1000 inhabitants per day from the available data of WHO’s GLASS project [18].

The threats posed by AMR are of increasing concern, even in low- and middle-income countries (LMICs), as the rates of antibiotic use increase. Assessing the extent of AMR in LMICs is complicated due to inadequate surveillance programs, though efforts like the WHO’s GLASS project (2015) have been initiated [18]. The misuse of antibiotics in humans, animals, and crops, along with the mismanagement of pharmaceutical waste during production, drives AMR in LMICs. Factors like poor sanitation, limited healthcare access, and lax regulations contribute to AMR spread. The COVID-19 pandemic compounded these challenges, increasing antibiotic misuse in LMICs and diverting attention from the AMR threat [19]. AMR hotspots are seen in parts of India, China, Pakistan, and other regions. Emerging resistance is noted in Kenya, Morocco, and Uruguay. Survey coverage did not always match hotspots. Common antimicrobial classes in animal production had high resistance rates [20]. Through the active participation of various countries in the WHO’s GLASS project, valuable insights have been gleaned regarding the total consumption of antibiotic, measured in terms of Defined Daily Doses (DDD) per 1000 inhabitants per day [18] (Figure 2). However, it is important to acknowledge that these findings are limited due to the lack of universal public engagement in the specific country data collection process.

Treating antibiotic resistant infections usually requires the prolonged use of last-line antibiotics, and these approaches often fail, contributing to infection persistence [21]. Several factors contribute to the development and spread of antibiotic resistance [22], and among these are:

- Overuse and Misuse of Antibiotics: The over-prescription and inappropriate use of antibiotics, such as using them to treat viral infections, contribute to the development of resistance [23,24,25,26,27].
- Incomplete Treatment: When individuals do not complete their prescribed antibiotic course, it creates an environment for the survival of the resistant bacteria, leading to the proliferation of resistant strains.
- Use in Agriculture: Antibiotics are extensively used in livestock and agriculture to promote growth and prevent infections in animals. This widespread use contributes to the development of resistant bacteria that can be transmitted to humans through the food chain. In addition, mobile elements conferring antibiotic resistance can move from livestock-associated lineages to human lineages [28,29].
- Global Travel and Trade: Resistant bacteria can spread quickly across borders through international travel and trade, making antibiotic resistance a global issue [30].
- Lack of New Antibiotics: The development of new antibiotics has slowed significantly in recent decades, reducing the arsenal of drugs available to combat resistant infections [31].

For all these reasons, antibiotic resistance has become a global health crisis, affecting people of all ages and in all parts of the world and, according to the World Health Organization (WHO) [32], antibiotic resistance is one of the most significant threats to global health, food security, and development. In this regard, the Centers for Disease Control and Prevention (CDC) estimate that, in the United States alone, more than 2.8 million antibiotic-resistant infections occur each year, resulting in over 35,000 deaths [33].

Moreover, antibiotic resistance has profound consequences on global health such as in: (i) difficult-to-treat infections; (ii) increased morbidity and mortality; (iii) hospital-associated infections; (iv) animal health impact; and (v) environmental and food safety impact.

CDC listed four threats in 2020 as being of urgent concern in the AMR [34] (Table 1):

- Carbapenem-resistant *Acinetobacter*
- Clostridioides difficile
- Carbapenem-resistant Enterobacterales (CRE)
- Drug-resistant Neisseria gonorrhoeae

Furthermore, the following were listed as serious threats highlighting the alarming global impact of antibiotic resistance and its implications for public health: Vancomycin-Resistant Enterococci (VRE), Extensively Drug-Resistant Tuberculosis (XDR-TB), and Methicillin-Resistant *Staphylococcus aureus* (MRSA) [34].

Addressing these issues requires a concerted effort from governments, healthcare providers, researchers, and the public to promote responsible antibiotic use, invest in the research and development of new treatments, and improve infection prevention and control measures.

## 2. Antibiotic Resistance: A Threat to Human and Animal Health

Over the past few decades, the indiscriminate and inappropriate use of antibiotics has led to the emergence of increasingly resistant bacterial strains, rendering previously effective drugs ineffective in treating infections [3]. This phenomenon poses a significant challenge to modern medicine as it compromises the ability to treat a wide range of infectious diseases, putting the lives of millions of people and animals at risk, and demanding immediate and concerted action to address this critical global challenge. Because of the interconnectedness of human and animal health, a unified approach known as One Health, which recognizes the inextricable link between human, animal, and environmental health, is needed [35] (Figure 1).

Diseases that were once easily treatable with antibiotics, such as pneumonia, urinary tract infections, and skin infections, now become formidable adversaries, necessitating more aggressive and costly treatment approaches. The loss of effective antibiotics not only jeopardizes the treatment of common infections but also undermines critical medical procedures such as surgeries, organ transplants, and cancer treatments, where the availability of effective antimicrobial agents is essential [36,37]. Similarly, animal health is significantly impacted by antibiotic resistance. In veterinary medicine, the diminished efficacy of antibiotics against resistant bacteria poses challenges in treating and controlling infectious diseases in livestock, companion animals, and wildlife [38]. This not only compromises animal welfare but also raises concerns regarding zoonotic diseases, where antibiotic-resistant bacteria can be transmitted from animals to humans. Livestock-associated antibiotic resistance can enter the food chain, leading to the consumption of antibiotic-resistant bacteria through animal-derived food products, further contributing to the spread of resistance and posing risks to human health [39,40,41].

This interplay between human and animal health cannot be overlooked. The data indicate that, among the countries presently utilizing veterinary antibiotics, five nations are predicted to experience the most significant percentage growth by 2030. These countries are Myanmar and Indonesia, followed by Nigeria and Peru, with Vietnam also showing a moderate increase [20]. To effectively combat antibiotic resistance, collaboration is paramount among stakeholders from various sectors, including healthcare professionals, veterinarians, researchers, policymakers, and the public. This is essential to optimize animal care, reduce the selection pressure for antimicrobial resistance, and ensure continued access to vital antimicrobial agents. Resolving this complex issue does not have straightforward solutions, but veterinarians must carefully consider the implications of their daily decisions and strive to optimize antimicrobial use for the benefit of their patients and society. Robust surveillance systems should be established to monitor resistance patterns in both human and animal populations, allowing for early detection and response [42]. Several studies have shown that aquatic bacteria resistant to specific antibiotics commonly found with *E. coli*, a human pathogen, shared some genetic elements and determinants with human and animal bacteria [43,44]. Responsible antibiotic use must be promoted, emphasizing appropriate prescribing practices in healthcare settings, and implementing guidelines for antibiotic use in agriculture. Eliminating human antibacterial use can potentially result in a more significant reduction (65.7–99.7%) in the colonization of resistant bacteria over 20 years [45]. On a smaller scale, reducing human-to-human transmission has the potential to reduce the AMR by 8.2–36.3% [45]. Strengthening infection control measures in healthcare facilities and animal farming is crucial in minimizing the spread of resistant bacteria [46]. Furthermore, substantial investment in research and development is necessary to drive innovation in the field of antibiotics. Efforts should focus on discovering new antibiotics, exploring alternative treatment strategies, such as phage therapy, nanoparticles, probiotics, and immunotherapies, and developing rapid diagnostic tools to facilitate targeted treatment. Education and awareness campaigns play a pivotal role in fostering a greater understanding of antibiotic resistance among healthcare providers, veterinarians, policymakers, and the public. By promoting behavioral changes that prioritize judicious antibiotic use and infection prevention, we can collectively combat the spread of antibiotic resistance. The challenges posed by antibiotic resistance demand a multifaceted and sustained effort. International collaboration is crucial in sharing knowledge, resources, and best practices to address this global crisis. Additionally, policy interventions are necessary to regulate the use of antibiotics in both human and animal health sectors, ensuring their prudent and responsible use. Investments in research and development should be incentivized to encourage the discovery of new antimicrobial agents and the development of innovative therapeutic approaches [47].

A recent analysis investigated the direct factors contributing to the AMR in both humans and food-producing animals on a global scale. Bidirectional associations were found: (i) animal antibiotic consumption was positively linked with resistance in critical priority human pathogens (1.07 [1.01–1.13]; *p* = 0.020) and (ii) human antibiotic consumption was positively linked with animal AMR (1.05 [1.01–1.09]; *p* = 0.010) [48]. AMR rates are significantly associated with antibiotic consumption in both humans and animals. Socioeconomic factors like income inequality, gross domestic product, and governance quality are linked to AMR. Different regions exhibit varying rates of AMR, with low- and middle-income countries (LMICs) often showing higher resistance rates compared to high-income countries (HICs) [48].

By embracing the principles of the One Health concept and working collaboratively, it is possible to mitigate the impact of antibiotic resistance, safeguard the effectiveness of antibiotics, and secure a healthier future for both humans and animals. Through responsible antibiotic use, improved infection control practices, surveillance systems, and ongoing research and development, we can collectively combat antibiotic resistance and preserve the efficacy of these life-saving medications for generations to come, if socio-economic country-specific factors are taken into account [42]. 

## 3. Combating Antimicrobial Resistance: A One Health Approach for Global Health Security

Due to the increasing development of multidrug-resistant bacteria, which renders existing antimicrobials ineffective in treating illnesses caused by these bacteria, AMR is a major global concern [4]. This predicament emphasizes the critical need for innovative treatments. Ineffective antimicrobials have serious effects, including increased mortality rates, as antimicrobial-resistant microbes grow increasingly common [37]. Addressing AMR necessitates a holistic approach known as One Health, which entails creating, implementing, and monitoring AMR surveillance programs, policies, and research. To achieve improved public health results, the One Health strategy encourages intersectoral collaboration among the public health, animal health, and environmental health sectors. Antibiotic stewardship is important in the One Health paradigm because it emphasizes ethical antibiotic use among healthcare providers, veterinarians, and farmers [49]. Adhering to antibiotic-use guidelines and policies helps to lessen the selective pressure that develops antibiotic resistance. Another important feature of the One Health strategy is interdisciplinary research, which allows for a thorough knowledge of the complex dynamics of antibiotic resistance [35]. By incorporating findings from a variety of domains, including human and veterinary medicine, ecology, microbiology, and others, it will be possible to obtain a thorough picture of how antibiotic resistance develops, spreads, and can be tackled by merging knowledge from several areas. This comprehension drives the development of novel tactics such as new diagnostic tools, alternative therapies, and vaccinations. The One Health approach also argues for comprehensive policies to be implemented at the national and international levels [35]. While some antimicrobial classes, such as those used to treat tuberculosis or illnesses uncommon in animals, are primarily designated for human usage, most antimicrobial classes are utilized in both people and animals. This includes the use of these chemicals in domestic mammals, birds, farmed fish, and shellfish, honeybees, and other creatures [50,51,52,53,54]. Certain antimicrobials are used in horticulture on occasion to treat and prevent bacterial diseases in fruits, such as “fire blight” in apples and pears caused by *Erwinia amylovora* [35]. Antimicrobials are used in human medicine to treat clinical infections in patients, with limited prophylactic usage for individuals or groups (e.g., post-surgery or post-exposure prophylaxis for vaccine preventable diseases). Antimicrobial use differs significantly between companion animals (e.g., dogs, cats, pet birds, horses) and food-producing animals, according to veterinary medicine [55]. Companion animals receive antimicrobial treatments in a manner comparable to humans. Antibiotics are predominantly prescribed for individual animals to address clinical illnesses, with occasional preventive use, such as after surgeries [56]. When some animals in a group require treatment for clinical infection, antimicrobial therapy is frequently delivered to the entire group through feed or water, even if most animals show no indications of infection. This method, recognized in the animal health industry as “therapeutic” use, varies from therapeutic use in human medicine. Furthermore, antimicrobials are used in food animals to treat clinically unwell animals, such as dairy cows with mastitis. The term “metaphylaxis” refers to group-level therapeutic and/or preventative treatment that includes the mass delivery of therapeutic doses of an antibiotic to a high-risk group of animals [57]. Because of the increased risk of bovine respiratory disease, one example of metaphylaxis is the administration of injectable antimicrobials to groups of calves upon their arrival at a feedlot. Thus, adopting a One Health approach is crucial in AMR for the sake of global health security. The rapid spread of multidrug-resistant bacteria and the ineffectiveness of existing antimicrobials highlight the urgent need for innovative treatments. The One Health approach places antibiotic stewardship at its core, recognizing its vital importance and direct impact on reducing antibiotic resistance [58,59]. Antibiotic stewardship programs constitute a fundamental component of preventive strategies. These programs aim to optimize antibiotic use by promoting judicious practices to mitigate the development of resistance by: (i) ensuring that antibiotics are employed only when absolutely necessary, (ii) selecting the most appropriate antibiotic for each specific situation, and (iii) administering it in the proper dosage for the appropriate duration [60]. This approach centers on responsible antibiotic use among various stakeholders, such as governments, healthcare providers, veterinarians, researchers, pharmaceutical companies, and farmers. By adhering to guidelines and policies pertaining to responsible antibiotic usage, we can reduce the selective pressure that drives antibiotic resistance. This stewardship should be embraced across all the involved stakeholders and be accompanied by educational campaigns and robust policy frameworks to support its successful implementation. In more detail, global initiatives, such as the World Health Organization’s Global Antimicrobial Resistance Surveillance System (GLASS) (https://www.who.int/initiatives/glass, accessed on 15 July 2023), demonstrate the global effort to combat antibiotic resistance and the importance of international cooperation. The national action plan for combating antibiotic-resistant bacteria, developed by US Department of Health and Human services (HHS), is a 2020–2025 plan [61] including the One Health approach as effective plan. The first objective is to identify and implement measures to foster stewardship of antibiotics in animals, eliminate the use of medically important antibiotics for growth promotion in animals, and bring under veterinary oversight other uses of medically important antibiotics. By year 5, new regulations were already implemented and antibiotic drugs used for feed transitioned from over-the-counter to Veterinary Feed Directive (VFD) or prescription status [62]. Likewise, the USDA and FDA collaborated to create, execute, and assess educational outreach efforts aimed at promoting responsible antibiotic use and stewardship. This involved interacting with livestock and veterinary associations to engage relevant stakeholders and ensure effective communication [62].

## 4. Collaborative Strategies for Tackling Antibiotic Resistance

Antibiotic resistance is an urgent worldwide health problem that endangers the well-being of both humans and animals. This challenge stems from the improper and excessive use of antibiotics in diverse fields, resulting in the emergence of bacteria that are resistant to multiple drugs. Undoubtedly, it is a major cause for concern due to its correlation with elevated mortality rates and fears of regressing to a pre-antibiotic era. Additionally, it has the potential to hinder efforts in controlling infectious diseases, to escalate healthcare expenses, and to undermine the progress achieved in healthcare [63]. Tackling this crisis requires a comprehensive and collaborative strategy that goes beyond the conventional barriers of disciplines and sectors.

The implementation of the One Health approach plays a pivotal role in effectively addressing antibiotic resistance [35]. The One Health approach recognizes the interdependence of human, animal, and environmental health, and highlights the importance of collaboration across several sectors including scientific, social, and political ones. To this end, it promotes the establishment, execution, and oversight of comprehensive surveillance programs, policies, and research initiatives to combat antibiotic resistance. Indeed, by promoting both worldwide cooperation and coordination among public health officials, veterinarians, environmental scientists, and policymakers, the One Health approach fosters a unified front to tackle antibiotic resistance.

In addition, the implementation of policies, both at national and international levels, is an essential element in addressing antibiotic resistance. It is, in fact, crucial to establish comprehensive policies that effectively regulate the use of antibiotics in humans, animals, and the environment, to mitigate the factors that contribute to antibiotic resistance. These policies should be developed collaboratively with stakeholders from all pertinent sectors, ensuring diverse perspectives are considered. They must be adaptable and flexible, capable of incorporating new scientific discoveries and adjusting to evolving circumstances. By prioritizing policy implementation, we can then foster a concerted and proactive response to combat antibiotic resistance. The global approach to antimicrobial resistance surveillance, in line with the One Health concept, acknowledges the complexity of the issue [64,65]. However, it is important to note that One Health surveillance should not necessarily mean integrating all surveillance components into a single system. ABR surveillance involves multiple sectoral components collecting diverse data for specific objectives. These components have distinct priorities, expectations, and limitations. To establish an effective One Health surveillance system, it is crucial to align sectoral components around a common objective, defined collectively with stakeholders. Collaboration should enhance data harmonization without compromising sector-specific goals [65]. International organizations can aid countries in creating suitable governance models and identifying necessary cross-sector collaborations based on their national surveillance context. The transition towards a One Health surveillance system is being carried out in various instances globally [65,66,67,68,69,70,71,72,73,74,75,76,77,78,79,80,81,82].

Promoting education and raising awareness constitutes another fundamental aspect of the strategy to combat antibiotic resistance [83]. Providing education and awareness to the public, healthcare professionals, and farmers regarding the prudent use of antibiotics and the associated risk of misuse is essential. This approach encourages responsible behavior, reduces instances of antibiotic misuse and overuse, and fosters a culture of antibiotic stewardship [29]. Notably, the European Union has taken significant steps, including a ban on nonmedicinal antibiotics in animals, while the USA has implemented more stringent antibiotic regulations similar to hospital antibiotic stewardship programs. Additionally, some regions have adopted policies mandating veterinary oversight and prescription for antibiotic use in livestock [29]. Furthermore, there are policies in place that restrict the use of specific antibiotic classes critical for human medicine in agriculture, such as fluoroquinolones and third-generation cephalosporins, in order to safeguard their effectiveness in treating human infections [84,85]. The zoning and separation of livestock production from human communities are also considered in some areas as measures to prevent the direct transmission of resistant organisms between animals and humans.

In conclusion, addressing antibiotic resistance necessitates a unified, collaborative, One Health approach that engages stakeholders from various disciplines. By embracing antibiotic stewardship, interdisciplinary research, comprehensive policy implementation, and education, we can effectively combat the factors driving antibiotic resistance and safeguard the well-being of humans and animals. This cooperative strategy is crucial for global health security and the long-term sustainability of human and animal health, demanding continuous dedication and investment from all sectors of society.

## 5. Prospective and Prevention Strategies

To address the growing problem of antibiotic resistance, it is crucial to adopt innovative, forward-thinking, and preventive approaches that encompass the interconnected realms of human, animal, and environmental well-being. Anchored in the One Health approach, these strategies should aim to proactively address the rise and dissemination of antibiotic-resistant bacteria, ultimately ensuring the long-term efficacy of antibiotics for future generations. The identified key areas of focus, in line with the One Health approach and aiming to address critical global challenges, are:

1. One Health Governance
2. Comprehensive Surveillance Systems
3. Stewardship Programs and Education
4. Research and Development
5. Preventive Measures
6. Public Education and Awareness

“One Health Governance” encompasses the formulation and administration of policies, frameworks, and mechanisms aimed at fostering a united and harmonized approach to addressing health challenges that intersect human, animal, and environmental health. This concept aptly acknowledges the interdependencies among these spheres and underscores the imperative for cross-sectoral and interdisciplinary endeavors to competently manage and avert health hazards that go beyond the conventional confines [86]. The academic community has the potential to champion the establishment of a worldwide framework for One Health governance, which would furnish guidance and provide benchmarks for nations aspiring to bolster their own governance systems. This framework would facilitate the synchronization of endeavors, enhance communication channels, and guarantee uniform actions to confront health issues demanding a cooperative stance [86,87,88].

To effectively construct this framework, it is imperative to prioritize public participation, engaging a spectrum of stakeholders including communities, healthcare professionals, scientists, policymakers, and industries, in decision-making processes pertaining to health matters. Additionally, adhering to a consensus-driven approach in accordance with global standards, and ensuring that policies and interventions are designed to cater to the needs and rights of all individuals and communities, regardless of their socio-economic or geographical contexts, holds paramount importance in both LMICs and high-income countries.

An essential aspect involves the development and improvement in comprehensive surveillance systems, such as The National Antimicrobial Resistance Monitoring System (NARMS) (https://www.cdc.gov/narms/index.html, accessed on 15 July 2023) established by the Food and Drug Administration, the Centers for Disease Control and the US Department of Agriculture. These systems, in turn, would play a vital role in monitoring the emergence, spread, and impact of antibiotic-resistant bacteria, providing invaluable data to influence policy decisions, guide research activities, and enable the prompt identification and containment of potential outbreaks. For these reasons, strengthening reporting mechanisms and promoting data sharing at both national and international levels are crucial in reinforcing the effectiveness of surveillance efforts and fostering a unified global response to antibiotic resistance.

In this context, ensuring adherence and efficiency requires the establishment and implementation of stewardship programs throughout all sectors, ranging from human health and animal health to agriculture. These efforts should be reinforced by continuous education and training initiatives, as outlined in the National Plan. Local successes can serve as valuable insights for distilling a triumphant global initiative.

We must stress that investing in the research and development of novel antibiotics and alternative therapies is a necessity in our fight against antibiotic resistance. Indeed, given the complex and dynamic nature of antibiotic resistance, it is essential to diversify our tools against bacterial infections. This requires not only the development of new antibiotics, but also the exploration of alternative therapies such as bacteriophage therapy, immunotherapies, and probiotics. Additionally, research activities should also prioritize the development of rapid diagnostic tests capable of quickly detecting bacterial infections and accurately identifying their antibiotic resistance profiles. By doing so, we can enable more precise and effective treatment. Bioinformatic tools, and curated AMR reference databases in accurately predicting AMR from genome sequences, have a huge impact in genomic surveillance [89,90,91,92]. Currently, whole-genome sequencing (WGS) is a powerful alternative or combination for antibiotic susceptibility testing (WGS-AST). Different approaches include the simple presence or absence of known resistance genes and single-nucleotide polymorphisms (SNPs), as well as more advanced methods using machine learning and statistical models [89,90,91,92,93]. Metagenomics techniques have a key role in expanding AMR surveillance to a One Health framework, allowing for a more comprehensive understanding of the *resistome* across different reservoirs [89,90,91]. Genome-based studies will also facilitate the identification of new drug targets that can be used for developing novel antimicrobial agents and therapies [89,90,91,92,93].

Moreover, it is necessary to prioritize preventive measures such as infection prevention and hand hygiene in healthcare settings, PPE and hand hygiene for bacterial diseases that are often associated with antibiotic resistance, and safe food handling and preparation [94]. In fact, by preventing infections at an early stage, we can significantly reduce the need for antibiotic treatments. The active promotion and enforcement of these practices across all sectors and settings are thus essential to effectively mitigate the risk of infection and subsequent antibiotic use.

Lastly, public education and awareness initiatives play a pivotal role as preventive strategies. By increasing public comprehension of antibiotic resistance and advocating for responsible use of antibiotics, we can influence behaviors and reduce the demand for unnecessary antibiotics. These campaigns should target diverse audiences, ranging from the public to healthcare professionals, and should employ a variety of communication channels to maximize their reach and impact.

In summary, it is evident that prospective and preventive strategies are crucial in fighting against antibiotic resistance. By establishing robust surveillance systems, advocating for antibiotic stewardship, allocating resources to research and development, improving infection prevention, and raising public awareness, we can effectively mitigate the emergence and dissemination of antibiotic-resistant bacteria. These collective efforts will ensure the continued effectiveness of antibiotics for the well-being of both current and future generations.

## Figures and Tables

**Table 1:** Antimicrobial-resistant bacteria: threat estimate. Adapted from reference [34].

	Resistant Bacteria	2020 Threat Estimate and 2019–2020 Change
URGENT	Carbapenem-resistant Acinetobacter	7500 cases, 700 deaths. Overall: 35% increase * Hospital onset: 78% increase
	*Clostridioides difficile*	**
	Carbapenem-resistant Enterobacterales (CRE)	12,700 cases, 1100 deaths. Overall: Stable Hospital onset: 35% increase
	Drug-resistant *Neisseria gonorrhoeae*	**
SERIOUS	Drug-resistant *Campylobacter*	26% of infections were resistant, a 10% decrease,
	ESBL-producing Enterobacterales	197,500 cases, 9300 deaths. Overall: 10% increase * Hospital onset: 32% increase *
	Vancomycin-resistant Enterococcus	50,300 cases, 5000 deaths. Overall: 16% increase * Hospital onset: 14% increase *
	Multidrug-resistant *Pseudomonas aeruginosa*	28,800 cases, 2500 deaths. Overall: Stable * Hospital onset: 32% increase *
	Drug-resistant nontyphoidal *Salmonella*	14% of infections were resistant, a 3% decrease.
	Drug-resistant *Salmonella* serotype Typhi	85% of infections were resistant, a 10% increase.
	Drug-resistant *Shigella*	46% of infections were resistant, a 2% increase.
	Methicillin-resistant *Staphylococcus aureus*	279,300 cases, 9800 deaths. Overall: Stable * Hospital onset: 13% increase *
	Drug-resistant *Streptococcus pneumoniae*	**
	Drug-resistant Tuberculosis (TB)	661 cases, decrease.
CONCERNING	Erythromycin-resistant group A *Streptococcus*	**
	Clindamycin-resistant group B *Streptococcus*	**

* Changes are in rates, not comparisons of counts. ** Data delayed due to COVID-19 pandemic.

## Data Availability

Not applicable.
